# RACK1, A multifaceted scaffolding protein: Structure and function

**DOI:** 10.1186/1478-811X-9-22

**Published:** 2011-10-06

**Authors:** David R Adams, Dorit Ron, Patrick A Kiely

**Affiliations:** 1 Department of Chemistry, Heriot-Watt University, Riccarton Campus, Edinburgh EH14AS, UK; 2 Ernest Gallo Research Center, Department of Neurology, University of California, San Francisco, Emeryville, CA, USA 94608; 3 Department of Life Sciences, and Materials and Surface Science Institute, University of Limerick, Limerick, Ireland

**Keywords:** RACK1, WD-repeat, guanine nucleotide binding protein 2-like 1, (Gβ), heterotrimeric G-proteins, PKCβII, scaffolding protein

## Abstract

The Receptor for Activated C Kinase 1 (RACK1) is a member of the tryptophan-aspartate repeat (WD-repeat) family of proteins and shares significant homology to the β subunit of G-proteins (Gβ). RACK1 adopts a seven-bladed β-propeller structure which facilitates protein binding. RACK1 has a significant role to play in shuttling proteins around the cell, anchoring proteins at particular locations and in stabilising protein activity. It interacts with the ribosomal machinery, with several cell surface receptors and with proteins in the nucleus. As a result, RACK1 is a key mediator of various pathways and contributes to numerous aspects of cellular function. Here, we discuss RACK1 gene and structure and its role in specific signaling pathways, and address how posttranslational modifications facilitate subcellular location and translocation of RACK1. This review condenses several recent studies suggesting a role for RACK1 in physiological processes such as development, cell migration, central nervous system (CN) function and circadian rhythm as well as reviewing the role of RACK1 in disease.

## Introduction

### The WD-repeat family of proteins

**Figure 1:**
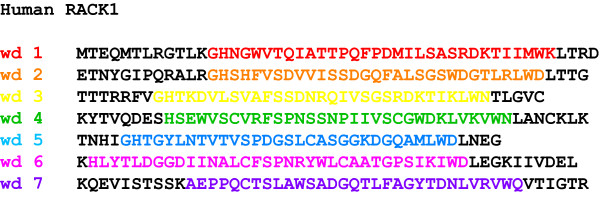
**Sequence of RACK1 depicting WD-repeats**. RACK1 is a 317 amino acid protein with seven 40 amino acid repeats which are defined by sequences flanked by GH and WD dipeptides.

**Figure 2:**
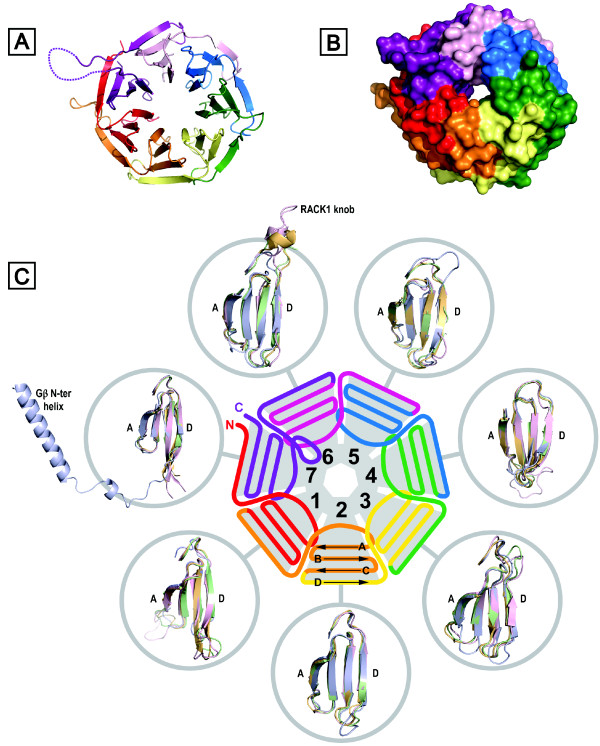
**Structural detail for RACK1 proteins**. (A) Crystal structure of RACK1A from *A. thaliana *(PDB: 3DM0), illustrating the seven-bladed β-propeller structure. (B) As (A) but with surface rendition. (C) Schematic representation of RACK1 structure and organisation of WD-repeats (defined in Figure 6); peripheral circles show superimposition of individual propeller blades for Gβ1 (blue) (PDB: 1TBG) and for the structurally defined RACK1 orthologues from *A. thaliana *(green) (PDB: 3DM0), *S. cerevisiae *(orange) (PDB: 3FRX) and *T. thermophila *(pink) (PDB: 2XZN). Gβ is distinguished in blade 7 by a helical N-terminal extension that engages tightly bound Gγ (not shown) in coiled coil interactions.

The tryptophan, aspartic acid repeat (WD-repeat containing proteins are an ancient conservative family of proteins found in prokaryotes and all eukaryotes [1]. They are involved in almost every signaling pathway and are associated with many genetic diseases. To date over 100 WD-repeat proteins have been assigned with an approved name and designation in the human nomenclature database. Genes encoding WD-repeat proteins are found on all chromosomes except 20, 22 (a pseudogene FBXW4P1 has been reported in chromosome 22), and the Y chromosome http://www.genenames.org This reflects their diversity and importance as a family, and suggests that their expression is regulated by a variety of signaling pathways. WD-repeats themselves are sequences of typically 44-60 amino acids in length ending at the C-terminus with a signature WD dipeptide or its equivalent (Figure 1). The motifs were first identified as repeating segments of homologous sequence within the primary structure of the transducin Gβ subunit and CDC4 [2]. Detailed analysis with a larger cohort of proteins revealed that the repeats are also typified by a characteristic GH dipeptide usually residing some 11-24 residues from the N-terminus, though neither the GH nor WD signature is absolutely conserved [3-5]. Several other characteristic amino acids contribute to the repeat, most notably an aspartic acid located 6 residues before the WD dipetide, but it is the collective critical mass of such features rather than the absolute conservation of any individual amino acid that establishes the identity of a sequence as a WD-repeat [6]. Given the variable number of residues at the N-terminal end of these units, sequence databases tend to map the repeats of WD-proteins between GH and WD dipeptides for convenience (Figure 1). The basic criterion for inclusion of a protein into the family is the presence of at least four of these repeat sequences to generate a WD-domain. These domains adopt a β-propeller structure, where the propeller fold is characterised by blades that are arranged radially around a central axis [3,7-10] (Figure 2). The conserved propeller structure is maintained by well-defined hydrogen bonding networks and intra-chain hydrophobic interactions (*vide infra*), although different WD-repeat proteins appear to adopt distinct folding orders for their constituent propeller blades [9,11]. In principle, a single β-propeller subunit may comprise four to eight blades [4,8], although at present only 7-bladed or 8-bladed WD-repeat propellers have been characterised by X-ray diffraction studies-the majority being 7-bladed structures consistent with the proposal that this is the optimal β-propeller fold [12]. Proteins are known with more than eight WD-repeats, but these assume tertiary structures with multiple propeller subunits (reviewed in [6]).

Although there is no single function for WD-repeat proteins, they share a common role in scaffolding protein complexes, often with multiple and competing partners, thereby serving as hubs for spatiotemporal orchestration of signaling events across diverse pathways. WD-repeat proteins themselves lack any enzymatic activity, but they are subject to post-translational modification, suggesting ways by which their cellular location and protein partnerships may be modulated. The lack of a direct catalytic enzymatic function for the protein family contrasts with other β-propeller-forming proteins, many of which exhibit enzymatic activity [8].

The most exstensively studied WD-repeat protein to date is the G-protein β subunit (Gβ) [7,13], which exists in a complex with the γ subunit (Gγ). Gβγ reversibly complexes with the α subunit to form a Gαβγ heteromer that associates with G-protein-coupled receptors (GPCRs) to enable transduction of extracellular signals. Upon ligand binding to a GPCR, the α subunit dissociates from the Gβγ heterodimer, resulting in the activation of various signaling cascades (reviewed in [14,15]). The mode of interaction of Gβ with binding partners has become increasingly clear with the emergence of crystal structures for Gβ in various complexes with Gα/Gγ, [9,10,16,17], phosducin [18-20], GPCR Receptor Kinase 2 (GRK2) [21-23], Regulator of G-protein Signaling 9 (RGS9) [24,25], and the Parathyroid Hormone 1 Receptor (PTH1R) [26]. Interestingly, WD-domains do not operate simply as standalone platforms for scaffolding protein complexes. Thus, several WD-proteins including RACK1 form both homodimers and heterodimers [27-29]. Moreover, some WD-repeat proteins contain other domains in addition to the WD-repeat sequences that increase the number of binding partners, scaffolding properties and overall function of the protein. For example, the β-transducing repeat-containing protein 1 (β-TrCP1) is a ubiquitin ligase with both WD-repeat and F-box domains as well as a RING domain. β-TrCP1 is required for the degradation of regulatory proteins such as Snail and p53 [30,31], and the WD-repeats increase its binding cohort and regulate its sub-cellular location, allowing the protein to also have a role in transcription and in regulating circadian rhythm [32-34]. WD-repeat and (suppressor of cytokines signaling) SOCS box-containing protein 1 (WSB1) is a ubiquitin ligase subunit, induced by Hedgehog signaling, that contains in addition to its WD-domain a SOCS-box that mediates its interaction with a ubiquitinating catalytic core complex [35,36]. WSB1 promotes cell survival in neuroblastoma [37]. Novel Nuclear Receptor Interaction Protein (NRIP) is a nuclear receptor with two WD-repeat domains which also has a nuclear localization signal [38]. NRIP interacts with the androgen receptor [39] and has recently been implicated in viral replication [40].

As our knowledge of the WD-repeat family of proteins expands, we are seeing that the members are involved in most signaling pathways (reviewed in [1]). It is no surprise, therefore, that WD-repeat proteins play a critical role in various human diseases. Subtle changes in the expression levels of WD-proteins can have a dramatic effect on protein complex assembly and on key signaling pathways. In this review, we focus on RACK1, a 36-kDa protein with seven WD-repeats that is homologous to Gβ, and that is one of the best known and studied members of the WD-repeat protein family. We focus on its structure, binding partners, subcellular localization, its role in signaling pathways and its function in normal physiological responses as well as in pathological conditions.

### RACK1 is a scaffolding protein

**Figure 3:**
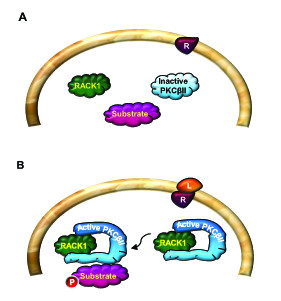
**Model for PKCβII and RACK1 interaction**. (A). In resting state, there is no interaction between RACK1 and inactive PKCβII. (B). Activation of PKCβII leads to the interaction between the active form of the kinase and RACK1. RACK1 then shuttles the active PKCβII to the site of its substrate.

**Figure 4:**
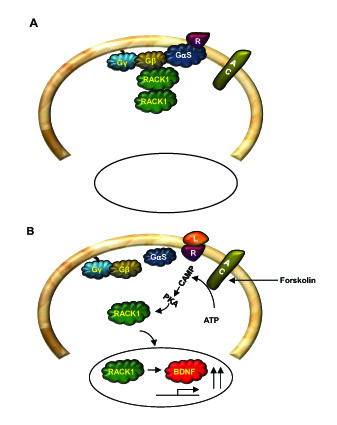
**Model for cAMP/PKA-mediated nuclear translocation of RACK1**. (A). In resting state, RACK1 forms homodimers as well as heterodimers with Gβ. (B). Activation of the cAMP/PKA pathway leads to the dissociation of RACK1 from the complex and its translocation to the nucleus. In the nucleus RACK1 contributes to cAMP/PKA mediated increase in BDNF transcription.

**Figure 5:**
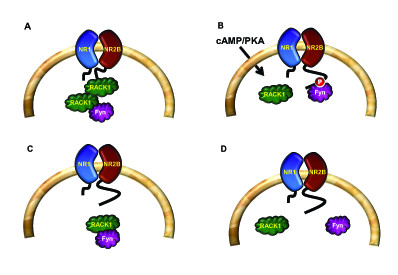
**Differential compartmentalization of RACK1 in the brain**. (A). In hippocampal and dorsal striatal neurons, RACK1 is associated with both the NR2B subunit of the NMDAR and Fyn kinase. (B). cAMP/PKA activation or alcohol exposure leads to the release of RACK1 from the complex, enabling Fyn to phoshorylate the tyrosine residues on the cytoplasmic tail of the NR2B. (C). In the cerebral cortex, RACK1 is bound to Fyn but not to the NMDAR. (D). In the ventral striatum, RACK1 is not associated with either protein.

RACK1 is a highly conserved intracellular adaptor protein with significant homology to Gβ. RACK1 was originally cloned from a chicken liver cDNA library and human B-lymphoblastoid cell line (B-LCL) [41]. Several years later, Mochly-Rosen's group cloned the protein from a rat brain c-DNA library which was screened for gene products that bind purified rat brain PKC in the presence of its activators (phosphatidylserine, diacyglycerol and calcium) [42]. Given the association of RACK1 with the active conformation of PKCβII, the protein was named Receptor for Activated C Kinase 1 (RACK1) [42-45] (Figure 3). However, it is now well established that RACK1 interacts with a large number of proteins either directly or as part of a larger complex. As a scaffold protein, RACK1 integrates inputs from distinct signaling pathways and is critical for fundamental cellular activities such as cell proliferation, transcription and protein synthesis, as well as various neuronal functions. RACK1's scaffolding properties are mediated by the presence of seven WD-repeats [46,47] (see also sections 2.2 and 2.3) that present multiple protein-binding sites and facilitate interaction with specialized protein docking modules, including SH2 domains (Src and Fyn) [48,49], plextrin homology (PH) domains (dynamin and p120GAP) [50,51] and C2 domains (PKCs) [43,45]. In addition, RACK1 was pulled out in a yeast two-hybrid screen as a binding partner of the first PDZ domain of the human Na^+^/H^+ ^exchanger regulatory factor (NHERF1) [52]. RACK1 also forms a heterodimer with at least one other WD-repeat protein, Gβ [27,28,53]. The formation of the RACK1-Gβ heterodimer has been shown to enable efficient cross-talk between signal transduction pathways mediated by GPCRs and by ligand-gated ion channels, specifically between the cAMP/PKA pathway and the N-methyl D-aspartate receptor (NMDAR) [29] (Figures 4 and 5). RACK1 also functions as a homodimer enabling the expansion of its binding partners [28,29,49,53,54]. For example, the formation of a homodimer enables the simultaneous interaction of RACK1 with Fyn and with the NR2B subunit of the NMDAR, even though Fyn and NR2B share the same binding site on RACK1 [29,49,55-57] (Figure 5A).

The proteins interacting with RACK1 fall into two broad categories, those constitutively bound and those that are stimulus-dependent or transient. RACK1 binding partners have been identified at various locations throughout the cell, and one important functional role for RACK1 is undoubtedly to shuttle some of these binding partners to particular intracellular sites. However, in addition to this role, a key aspect of the function of RACK1 is its capacity to modulate the enzymatic activity of its binding partners, either promoting or suppressing the activity of bound enzymes. For example, RACK1 stabilizes the activity of protein phosphatase 2A (PP2A) [58,59] and maintains the active conformation of PKCβII [42,45,60] (Figure 3). In contrast, RACK1 binds to the tyrosine kinases Src and Fyn to maintain these kinases in an inactive state until the appropriate signal occurs to trigger dissociation of the complex [29,49,55-57,61,62] (see also Figure 5A). Thus, RACK1 plays a key role in stabilizing the active or inactive conformation of its partners.

## RACK1, Gene and Structure

### RACK1: Gene, promoter and expression

**Figure 6:**
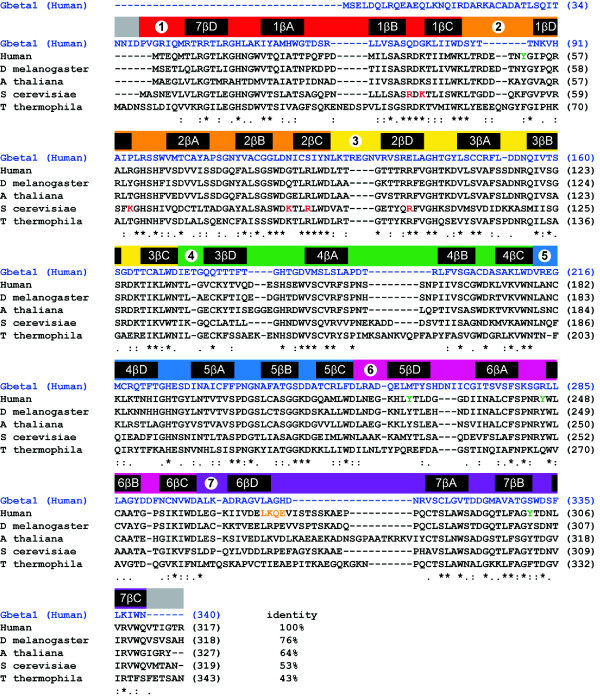
**Sequence alignment of Gβ1 (UniProtKB accession ID: P62873) vs RACK1 orthologues from Homo sapiens (P63244), *Drosophila melanogaster *(O18640), *Arabidopsis thaliana *(O24456), *Saccharomyces cerevisiae *(P38011) and *Tetrahymena thermophila *(E6PBV0)**. This alignment is similar to that presented by Ullah *et al *[81] but revised to include the sequence of the T. thermophila protein. WD-repeats and secondary structural elements are defined above the sequences. The percentage sequence identity between human RACK1 and the orthologues from other species is defined at the end of the alignment. The homology index for the proteins (excluding Gβ1) is presented below the sequence, where fully conserved residues are marked with an asterisk; conservative and semiconservative positions are marked with colons and periods respectively. Residues marked in red in the yeast sequence engage rRNA in the eukaryotic 40S ribosomal subunit (see text).

Human RACK1 is encoded by the gene, *GNB2L1 *[guanine nucleotide binding protein (G-protein), beta polypeptide 2-like 1], which has 8 exons and 7 introns [63,64]. The open reading frame of the gene is 1142 bp and it encodes for a protein with 317 amino acids, registering as a 36 kDa protein on an SDS PAGE gel. Studies probing the evolution of human RACK1 suggest that human, rat and mouse RACK1 form a different subgroup and are therefore strictly paralogs of RACK1 in other organisms, but these studies nevertheless indicate that RACK1 is strongly conserved through evolution [64]. Indeed, sequence alignments of RACK1 species from diverse organisms -- *Tetrahymena thermophila, Saccharomyces cerevisiae, Arabidopsis thaliana *and *Drosophila melanogaster *-- reveals a 43-76% sequence identity (Figure 6). *GNB2L1 *is mapped to chromosome 5q35.3, in close proximity to the telomere of chromosome 5 [63]. At least 6 other genes have been mapped to this region and it is a reported location for cytogenetic and molecular abnormalities [65-68]. The promoter region of *GNB2L1*, which was found to have two alternative start sites of transcription, has binding sites for cardiac/smooth muscle specific c-Rel (NF-κB) [63,69]. RACK1 is highly expressed in most tissues [70], including the brain [71]. The expression of RACK1 appears to be tightly regulated and changes in RACK1 expression are associated with cancer (see section 6.1.). It has also been suggested that changes in RACK1 expression contribute to brain pathologies [72-74] (see section 6.2.). This is supported by observations from our group and others that RACK1 is inhibitory to the growth of normal fibroblasts and can only be over-expressed in transformed cells or cells that over-express the IGF-1R [75-77]. Interestingly, a binding site for the glucocorticoid receptor is present in the *GNB2L1 *promoter [63], suggesting a mechanism by which RACK1 expression levels may change with aging. This is significant given that RACK1 affects the subcellular distribution and function of numerous proteins and, as a result, plays an essential role in regulating signaling pathways in many key biological processes [46,47]. Indeed, over 80 binding partners of RACK1 have been reported to date, (although several of these remain uncharacterised and may not bind to RACK1 directly), and as new partner proteins emerge, the important contribution that RACK1 makes to signaling pathways is becoming clearer. It is probable that RACK1 engages in different sets of signaling pathways in different cells, reflecting the cell type and the differential expression of its binding partners. In this way, RACK1 plays distinct roles in different cell types, even when the expression of RACK1 itself remains relatively constant.

### Structure of RACK1

Gβ was the first WD-repeat protein to be characterised by X-ray crystallography [9,10,16]. Numerous crystal structures have since emerged for WD-repeat proteins [9,10,78] and these now include recently determined structures for three RACK1 species: RACK1A from *A. thaliana*, Asc1p from *S. cerevisiae *and RACK1 from *T. thermophila *at 2.4, 2.1 and 3.9 Å resolution, respectively [79-82]. The *T. thermophila *protein structure was obtained as a complex with the 40S small ribosomal subunit from this protozoan. A structure for the yeast RACK1 orthologue was also determined in complex with the 40S ribosomal subunit at similar resolution (4.15 Å) [83]. These structural studies confirmed that RACK1 adopts a seven-bladed β-propeller structure consistent with the predictions of earlier homology modelling studies based on Gβ and other WD-repeat proteins [46,78-81]. The *A. thaliana *protein was the first of the three RACK1 orthologues to be structurally defined and, of the three, it exhibits the highest sequence homology to human RACK1, with 64% residue identity [81] (Figure 6). In contrast to metazoans, where RACK1 is expressed from a single gene, *A. thaliana *possesses three genes-*RACK1A*, *RACK1B *and *RACK1C *- for closely related proteins, with the sequences for RACK1B and RACK1C (not shown) exhibiting similar levels of homology to the human protein. The structure of RACK1A is shown in Figure 2A. Each propeller blade consists of a four-stranded antiparallel β-sheet, where strand A lines the central canal of the protein, a channel of ~9 Å width (Figure 2B), and strand D is presented on the outer circumference. Adjacent blades are connected by a loop bridging from strand D on one blade to strand A on the next. These loops are exposed on the top face of the propeller blade, as are the β-turns linking strands B and C in each blade. The loops connecting strand A to B and strand C to D in each blade are located on the reverse, slightly larger face of the propeller.

**Figure 7:**
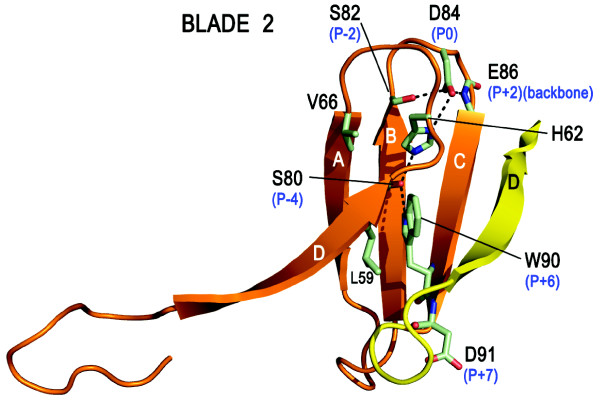
**Summary of conserved interactions in 'typical' WD-repeats responsible for maintaining the structural integrity of propeller blades 1-5 in RACK1**. Interactions are illustrated for blade 2 of the *A. thaliana *RACK1A structure (PDB: 3DM0). The second WD-repeat (as defined in Figure 6) is shown in its entirety (orange ribbon) and contributes β-strand D to blade 1 plus strands A, B and C of blade 2, terminating in the WD signature residues (W90-D91). Strand D of blade 2 is provided by residues in the third WD repeat (yellow ribbon). A highly conserved Asp residue (D84) occupies the central position of the B-C turn and is networked by hydrogen bonds to residues in the P-2 and P+2 positions (S82 side chain, E86 backbone) as well as through a salt bridge to the signature GH His (H62) in the D-A loop between blades 1 and 2. H62 is further networked with a hydrogen bond to S80 on β-strand B and a hydrogen bond between S80 and W90 (β-strand C). Important non-polar interactions regulate blade packing such as contact between W90 and residues on the reverse face of blade 1 (L59) and between V66 (β-strand A) and the reverse face of the B-C hairpin of blade 1 (not shown). B-C hairpin positions relative to the consensus Asp (P0) are marked in blue.

The WD-repeats of RACK1, as with all proteins that adopt this fold, overlap two adjacent propeller blades to provide an interlocking architecture. Each repeat encompasses the D-strand of one blade and strands A, B and C of the next, terminating in the signature WD dipeptide at the end of strand C such that the aspartic acid (or equivalent residue) is exposed on the propeller's lower face. The tryptophan, which is sometimes replaced by phenylalanine or tyrosine, is sandwiched between adjacent blades and plays an important role in inter-blade spacing (Figures 2 and 6). As the N-terminal sequence in the first WD-repeat contributes strand D of the seventh blade, hydrogen bonding to the strand C sequence of the last repeat, the assembly is sealed with a 'Velcro-like' fastening to complete the toroidal structure [5]. Superimposition of the available RACK1 crystal structures with human Gβ_1_, shown for individual propeller blades in Figure 2C, reveals that the greatest structural variation lies in the A-B, C-D and D-A loops, which are of variable length. Most notably, the D-A loop between blades 6 and 7 in the RACK1 species is 8-19 residues longer than the cognate region of Gβ_1 _and forms a pronounced knob-like projection from the upper face of the propeller (disordered in the crystal structure of *A. thaliana *RACK1A). In contrast to the three other loops, the central B-C hairpin of each blade adopts a tight β-turn with a strongly conserved backbone conformation. Some 85% of known WD-repeats possess an aspartic acid at the middle position of the three residues usually present in the B-C turn [11], and this highly conserved residue forms a salt bridge with the histidine of the glycine-histidine (GH) signature dipeptide (when present) as shown in Figure 7. This GH interaction plays an important role in defining the trajectory of the inter-blade D-A loop which hosts the GH dipeptide and therefore in stabilising the propeller structure. A network of additional hydrogen bonded interactions between strongly conserved residues at specific locations within the WD-repeat also maintains the integrity of blade structure. Thus, the histidine of the GH dipeptide is characteristically hydrogen bonded to a serine (or threonine) on β-strand B located in the P-4 position relative to the conserved aspartic acid (P-0) of the B-C turn. This serine is usually hydrogen bonded in turn to the indolic NH of the WD signature tryptophan (occupying the P+6 position). The structure of the B-C turn is additionally defined by an engagement between the P-0 aspartate side chain and the peptide backbone NH at the P+2 position (*i.e*. the first amino acid in β-strand C). The final residue in β-strand B (at the P-2 position) is invariably small (Ser, Gly or Ala) for typical blades^1 ^in order to accommodate the folding of the aspartic acid side chain. In the majority of instances the P-2 residue is serine, and in these cases the residue also engages the aspartate with a hydrogen bond to strengthen the local structure of the protein in that region. Non-polar interactions also play an important role in establishing the β-propeller structure however. For example, the opening residue in β-strand A that lies proximal to the B-C turn is almost invariably valine or isoleucine and fits into the space between the B- and C-strands of the preceding propeller blade, directly behind the P-0 aspartic acid of the B-C turn on that blade and regulating inter-blade spacing at that point. The WD signature tryptophan (or phenylalanine/tyrosine replacement) projects from the face of each propeller blade to pack against a bulky residue (frequently leucine) located at the end of the D-strand and projecting from the rear of the preceding blade.

**Figure 8:**
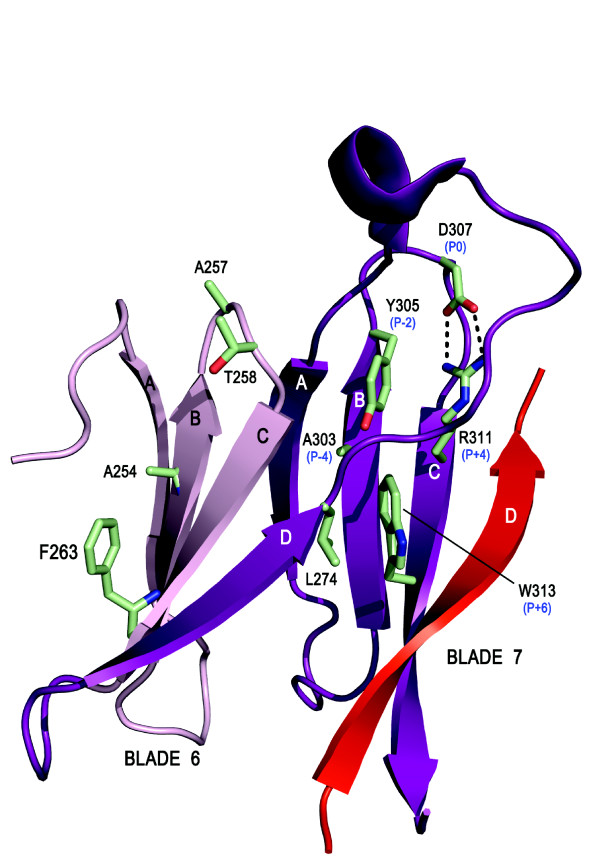
**Summary of key interactions in 'atypical' WD-repeats 6 and 7 of RACK1, illustrated with the structure of yeast Asc1p (PDB: **3FRX). RACK1 proteins are characterised by a significant sequence extension between blades 6 and 7, leading to a knob-like protrusion from the upper face of the propeller. The P0 Asp in blade 7 forms a salt bridge to an Arg at the P+4 position and this is packed against a Tyr (or in some orthologues Phe) in the P-2 position. The P0 Asp is absent in blade 6. In principle, these features together with the absence of the GH signature dipeptide on the loops between blades 5 and 6 and between blades 6 and 7 may facilitate structural transitions in this region of the protein that are important for function.

The features described above and summarised in Figure 7 are tightly conserved across blades 1-5 of the RACK1 proteins and are responsible for a highly regular and 'typical' organisation to the core of the propeller blades for this major segment of the proteins. Some notable deviations are seen in the core organisational elements for blades 6 and 7, however, and these are likely associated with the RACK1 sequence insert in the D-A loop between these two blades. This loop lacks the characteristic GH signature dipeptide to engage the conserved Asp of the B-C turn in blade 7. Instead, an Arg on β-strand C of blade 7 provides a surrogate salt bridge in lieu of the signature GH to stabilise the blade 7 B-C hairpin. This is illustrated in Figure 8 with the yeast RACK1 orthologue, where the D-A loop between blades 6 and 7 is of similar length to human RACK1 and fully ordered in the crystal structure. The cognate loop of RACK1 proteins in plants, as with *A. thaliana*, is significantly extended by comparison. The arginine residue (R311) that contributes the salt bridge to the B-C hairpin of blade 7 lies in the P+4 position relative to the aspartic acid (D307) and is packed against a tyrosine side chain (Y305) in the P-2 position of this atypical WD-repeat. In contrast, as noted above, the P-2 position is taken by Ser or Gly in typical WD-repeats (Figure 7). In blade 7 this rare occurrence of a Tyr is associated with the establishment of pi-cation interactions with the P+4 Arg (Figure 8). Significantly, the tyrosine is conserved across RACK1 orthologues of higher organisms and is a known phosphorylation site (*vide infra*) [58], suggesting a functionally important role for the atypical structure of this RACK1 WD-repeat. Indeed, the scaffolding of several proteins is known to be mediated by blades 5-7 of RACK1.

The D-A and B-C loops of WD-repeat proteins alternate with one another in sequence to generate the upper face of their β-propeller structures (as presented in Figure 2). Thus, the extended D-A loop that forms the RACK1 knob between blades 6 and 7 is followed by the B-C hairpin on blade 7 and preceded by the corresponding hairpin on blade 6. Given the atypical features just described in the B-C hairpin of blade 7, it is unsurprising that the corresponding hairpin of blade 6, immediately preceding the knob, should also differ from the typical consensus features summarised in Figure 7. The key distinction in this hairpin is the deletion of the central aspartic acid residue of the β-turn, and a very tight turn of two residues serves to reverse the backbone between β-strands B and C in blade 6, though neither of the residues at this site is conserved across RACK1 orthologues (Figure 6). In the yeast structure, the turn comprises A257 and T258 (Figure 8). Deletion of the aspartic acid from the B-C turn also appears to be associated with loss of the signature GH dipeptide from the preceding D-A loop between blades 5 and 6, where, in a typical WD-repeat, the histidine would engage the aspartic acid of the B-C turn. Thus, RACK1 proteins lack the GH motif in the D-A loops between both blades 5/6 and blades 6/7. Significantly, the absence of the GH in these loops appears to make the presence of the consensus P-4 serine on β-strand B redundant in blades 6 and 7, and the serine is replaced by alanine in both blades of yeast and mammalian RACK1 proteins. As shown in Figure 7, the P-4 serine hydrogen bonds to the preceding GH signature histidine in typical WD-repeats. Substitution of the P-4 serine by alanine has a further effect, however, and abolishes the additional hydrogen bond to the WD signature tryptophan that is seen in typical WD-repeats. The abolition of this hydrogen bond appears to be associated with a change in the conformation of the tryptophan side chain in blade 7 of the yeast protein, where the indolic NH is no longer constrained in a hydrogen bond towards strand B but re-orientated towards strand D instead. Interestingly, in blade 6 of the yeast structure, the WD tryptophan is replaced by phenylalanine, and it is conceivable that where the P-4 serine of the B-strand is substituted by a non-hydrogen bonding residue evolutionary conservation of the signature tryptophan is also relaxed.

### Structural basis for RACK1-scaffolded protein associations

The available crystal structures for Gβ and other WD-repeat proteins have established the structural basis for scaffolded interactions with a range of partner proteins-Gα/Gγ, phosducin, GRK2, RGS9 and PTH1R in the case of Gβ for example (*vide supra*)-and the current understanding of WD-repeat protein docking modes has recently been reviewed in detail [6]. Unfortunately, structural information relating specifically to the interactions of RACK1 with its numerous protein partners currently remains very limited.

Scanning peptide arrays, mutagenesis studies and competition experiments with RACK1-derived peptides have identified probable loci on the surface of RACK1 that may contribute to protein-protein interfaces for some of RACK1's partners. The most detailed information relating to RACK1-biomolecule associations currently available, however, has emerged from structural studies with ribosomes. Initial breakthroughs came with the use of cryoelectron microscopy (cryo-EM) techniques, affording a low resolution (11.7 Å) structure for the yeast 40S ribosomal subunit that identified the position of bound RACK1 on the head region proximal to the mRNA exit channel [84]. The cryo-EM map of a mammalian 40S subunit followed at slightly higher resolution (8.7 Å) [85]. These studies pre-dated the RACK1 X-ray crystal structures, and the ribosome structure was constructed by fitting homology models of individual ribosomal components (templated on Gβ in the case of RACK1) to the cryo-EM map. Both models implicated helices 39 and 40 of the 18S ribosomal RNA as the primary anchoring region for RACK1, with contact mediated largely by blades 1 and 2 and their associated loops. Consistent with this, binding of RACK1 to ribosomes is compromised by mutation of a number of basic residues that are conserved across the identified RNA binding interface of RACK1 species but that are absent at the cognate sites in Gβ (Figure 6). Thus, a double charge reversal mutation (R38D:K40E) in the RDK B-C turn of blade 1 almost fully ablates binding of the yeast protein (Asc1p) to the ribosome [79,84]. Alanine mutations for K87, R90 and R102, residues that project from the B-C turn and edge of blade 2 in Asc1p, also attenuate binding to the ribosome [79]. Similarly, alanine mutagenesis of K62, located on the edge of blade 1, weakens binding to the ribosome [79]. At least four of these residues (K40, K87, R90, R102) are expected to make direct contact with the sugar-phosphate backbone of the ribosomal RNA, forming salt bridge interactions with the phosphates. Interestingly, neither K62 nor K87 are fully conserved across RACK1 homologues, however, indicating some species differences in the interaction of RACK1 with the RNA of the 40S ribosomal subunit. Nevertheless, all the available data from structural studies with eukaryotic ribosomes points to a similar mode of engagement involving the edge of blades 1 and 2 and accompanying loops on the top face of the β-propeller. Interestingly, RACK1 associates with polysome bound polyA-mRNA enabling PKCβII to phosphorylate polyA-mRNA-associated proteins and control localized protein synthesis in neurons [86].

**Figure 9:**
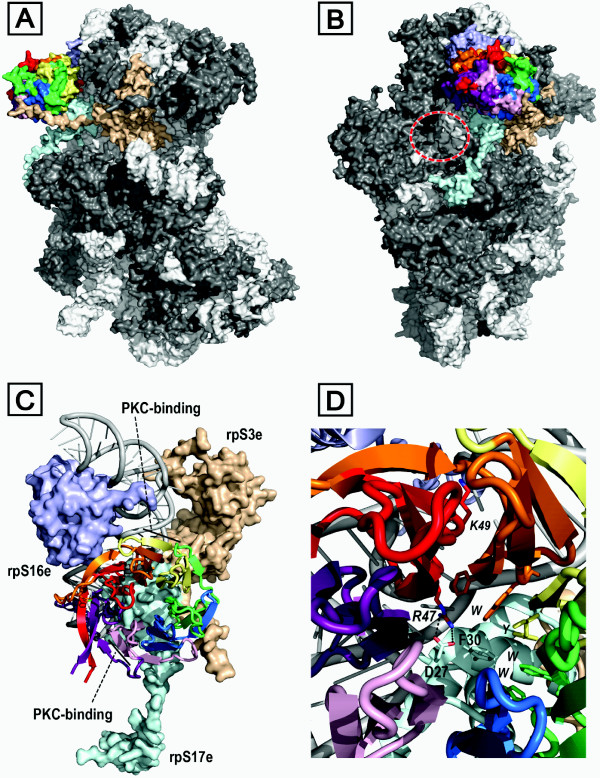
**Structure of RACK1 bound to the 40S eukaryotic ribosomal subunit and interaction with co-associated ribosomal proteins**. (A) Surface rendition of *T. thermophila *(PDB: 2XZN) 40S subunit: rRNA (white); RACK1 coloured as in Figure 2A/B; ribosomal proteins are shown in grey with the exception of those in direct contact with RACK1 -- rpS3e (light brown), rpS16e (light blue), rpS17e (pale cyan). (B) As (A) but with 90° rotation about the y-axis; position of mRNA exit tunnel is marked (dashed oval). (C) Detail of ribosome-bound RACK1 structure, highlighting PKC-binding loci on blades 2 and 6; RACK1 and rRNA are shown in ribbon format, interacting ribosomal proteins are surface-rendered. (D) Detail of key contacts between RACK1 residues (italicised) and rpS17e residues (non-italicised): D27 (rpS17e) forms a salt bridge with R47 (RACK1); F30 (rpS17e) packs against aromatic RACK1 side chains (W, Y, W, W). R47 (RACK1) engages rRNA backbone phosphates.

RACK1 binding to the eukaryotic ribosome is not purely mediated by interactions with the 18S RNA however. Additional contact occurs between RACK1 and co-associated ribosomal proteins and, following the studies described above, cryo-EM maps have emerged for ribosomes providing more detail for the assembled proteins [87-89]. Very recently X-ray crystal structures have also been obtained for complete eukaryotic ribosomal subunits [80,83]. These studies have defined the interfaces on RACK1 for co-association with three eukaryotic ribosomal proteins -- rpS3e, rpS16e and rpS17e -- as illustrated with the 40S subunit from *T. thermophila *at 3.9 Å resolution (Figure 9). The most extensive protein contacts occur with a three-helix bundle formed by the N-terminal region of rpS17e, which occupies one face of the RACK1 propeller. The model suggests that a number of salt bridges are formed at the interface between the two proteins, and these contacts involve the RDK turn between strands B and C of blade 1 in RACK1. Charge reversal mutations of the two basic residues in this conserved turn had earlier been found to abrogate binding of the yeast RACK1 ortholog (Asc1p) to the ribosome (*vide supra*). The additional detail provided by the crystal structure suggests that, whereas the Lys is orientated towards phosphates on the nearby rRNA backbone, the Arg of the RDK motif engages an Asp residue (D27) on rpS17e. A phenylalanine side chain (F30) on rpS17e, proximal to this Asp, inserts into the mouth of the RACK1 tunnel to form extensive packing interactions against aromatic side chains lining the inner rim formed by the RACK1 D-A and B-C loops. Contact between RACK1 and rpS16e is less extensive and focused almost entirely on the edge of blade 1. The third ribosomal protein, rpS3e, engages RACK1 with an extended C-terminal strand that runs diagonally across the edge of blade 4 and terminates in contact with the C-D loop of blade 5. This strand is not defined in a crystal structure for the yeast ribosome at 4.15 Å resolution and adopts a rather different trajectory across blades 4 and 5 in a model derived from a cryo-EM map of the ribosome at 6.1 Å resolution [83,89,90].

The ribosome binding mode for RACK1 established through the recent cryo-EM and X-ray diffraction studies indicates that the top face of the RACK1 propeller, as presented in Figure 2, is fully blocked when RACK1 is bound to the ribosome. In contrast, access to the reverse face of RACK1 and several of the blade edges is unobstructed, and these surfaces may serve as a platform for recruitment of proteins to the ribosome. One such protein known to be recruited to the translating ribosome is PKCβII [91-93]. Competition experiments with peptide sequences derived from WD-repeats 3 and 6 suggest that these regions of RACK1 contribute to the PKCβII binding interface [92,93]. Significantly, both loci are accessible in the established ribosome-bound RACK1 structures. Thus, a mammalian RACK1 sequence (99-RRFVGHTKDV-108) corresponding to the outer β-strand of blade 2 and D-A loop into blade 3 disrupts association of RACK1 and PKC [92]. This sequence, although located proximal to the rRNA of the 40S subunit (Figure 9), has significant surface exposure. Remarkably, sequences from WD6 that are implicated in PKC binding include both β-strands A (234-DIINALCF-241) and C (255-SIKIWD-260) of blade 6 [42-44,94-96]. Direct contact with residues in these strands can only reasonably occur by intercalation of structural elements from PKC between propeller blades 6 and 7 or, as has been proposed by others, [46] through the peeling off of the outer strands in blade 6 to allow hybridisation with surrogate strands from PKC. The sequence extension between blades 6 and 7 that forms the RACK1 knob may be important in providing flexibility for the structural transition required to accommodate PKC by either of these modes. This knob structure is bound close to rRNA and rp17e in the established 40S structures, but mutagenesis and sequence deletion studies indicate that it is not actually essential for binding of RACK1 to the ribosome, at least in the case of yeast [79]. Further work is required to provide a detailed structural understanding of the interaction between PKC and RACK1 and this should include consideration of the role of the RACK1 knob.

A very recent and exciting development in regards to RACK1 structure has been the acquisition of an X-ray crystal structure for the yeast protein, Asc1p, as a homodimer [82]. This new structure has revealed a previously unprecedented mode of β-propeller dimerisation involving blade hybridisation. Specifically, β-strands B and C in blade 4 are extruded from the upper face of the propeller as an extended loop for each of the protomers in the dimeric assembly. The remaining strands (A and D) from blade 4 stay within the propeller core but now hydrogen bond directly to one another. Thus, in effect strand D migrates radially inwards to take the position that the extruded strand B ordinarily occupies in the monomeric protein. The two RACK1 protomers then dimerise by hybridisation of the blade 4 D-strands, forming a shared four-stranded antiparallel β-sheet in the format, Protomer-A-D:D-A-Protomer. This mode of dimerisation also brings the blade 3 edges of each protomer sufficiently proximal to one another to hydrogen bond, forming an extended eight-stranded β-sheet. Remarkably, the extrusion-hybridisation mode of dimerisation centred on blade 4 causes little perturbation in the rest of the protein structure, and the protomers in the dimeric assembly superimpose tightly onto the structure of the monomeric RACK1 protein over the blades not directly involved in dimerisation. The emerging picture for the RACK1 homodimer provides important new insights into the potential interactions between RACK1 and its partner proteins. Firstly, it accounts for the capacity of RACK1 to simultaneously scaffold the NMDA receptor and Fyn (*vide infra*), where both of these proteins target overlapping binding loci on RACK1. Secondly, the model shows how buried sequences within the core propeller blades may potentially be exposed by extrusion to provide binding surfaces that are not exposed in the monomeric state. The model is particularly significant in relation to the association of PKC (discussed above), where one model for the interaction involves β-strand swapping between RACK1 and the β-sandwich C2 domain of the kinase. The RACK1 homodimer structure provides the first direct evidence that such strand extrusion-insertion mechanisms may indeed be important for the interaction of RACK1 with its partners, and, if this is applicable to the binding of PKCβII, that such mechanisms may occur with blades other than blade 4 alone.

## RACK1 and Signaling

### RACK1 and PKC

As detailed above, RACK1 was first described as a PKCβII anchoring protein [42], and has since been extensively studied in relation to PKC signaling [46,97]. RACK1 binds activated PKCβII and the interaction serves to stabilize the active conformation of the kinase [42,45,60] (Figure 3). In addition, RACK1 serves as a PKCβII shuttling protein, enabling the kinase to phosphorylate its substrate at the appropriate site [60] (Figure 2). Although most of the studies are focused on the interaction of RACK1 with PKCβII isoform, several other PKC isoforms bind RACK1 upon activation [46,98,99]. In addition, the RACK1/PKC partnership was suggested to be intrinsically linked and PKCs have been shown to regulate RACK1 expression in cardiac myocytes [100]. As well as stabilising PKC activity, RACK1 introduces activated PKC to new signaling proteins and pathways. These signaling proteins may be directly scaffolded by RACK1 or RACK1 may shuttle its cohort of bound PKC to particular cellular locations and anchor a subset of activated PKC at specific receptor sites. For example, RACK1 was shown to scaffold the MAP kinase (MAPK), Jun N-terminal kinase (JNK) and various PKC isoforms (αβγ) upon stimuli leading to PKC phosphorylation of JNK [101]. Thus, RACK1 allows the cross-talk between the PKC and MAPK pathways.

Recently, very exciting data published by the Lebel group suggests that depletion of Werner syndrome, RecQ helicase-like (WRN) protein in the nucleus causes RACK1 to move out of the nucleus and to collocate with PKCβII and PKCδ [102]. This suggests that PKC may also function as a 'sink' for RACK1 in the cytoplasm, preventing RACK1 from going to the nucleus non-specifically. The interaction between RACK1 and PKC family members in the nucleus has also been reported however. Specifically, RACK1 and PKCα are recruited in a circadian manner into a nuclear brain and muscle aryl hydrocarbon receptor nuclear translocator 1 (BMAL1) complex during the negative feedback phase of the cycle [103]. This finding suggests that PKCα is rhythmically activated during this process and that both RACK1 and PKCα are an integral part of the oscillatory mechanism. Finally, the interaction between RACK1 and PKC isozymes plays a role in development [104], as discussed in section 5.1.

### RACK1 and the cAMP/PKA pathway

The cAMP/PKA signaling pathway is also tightly linked to RACK1 [29,56,105-107]. Specifically, cAMP is hydrolysed through the action of cAMP phosphodiesterases (PDEs) [108], and RACK1 interacts with phosphodiesterase isoform PDE4D5 [107,109,110]. Interestingly, RACK1 and the scaffolding protein β-arrestin compete for binding to PDE4D5 [109]. Furthermore, and as detailed in section 5.2., RACK1 serves as the molecular bridge linking the Focal Adhesion Kinase (FAK) to PDE4D5 to control cell polarity [111,112]. In addition, we previously reported that upon activation of PKA by the adenylate cyclase forskolin, or the pituitary adenylate cyclase activating peptide (PACAP), RACK1 translocates to the nucleus where it mediates the expression of the brain-derived neurotrophic factor (*BDNF*) [56,106] (Figure 4). Finally, the activation of the cAMP/PKA pathway in hippocampal neurons also leads to the dissociation of RACK1 from its binding partners Fyn kinase and the NR2B subunit of the NMDAR, leading to the phosphorylation of the subunit by Fyn, and to enhancement of the channel's activity [49,56] (Figure 5A) (see also sections 3.3 and 3.4.2).

### RACK1 and the Src family of non-receptor protein tyrosine kinases

RACK1 plays an inhibitory role with the Src family of non-receptor protein tyrosine kinases (PTKs), specifically with Src and Fyn. Thus, RACK1 interacts with Src and Fyn respectively in cultured cancer cells and in neurons, and this interaction inhibits the activity of the kinases, suggesting that RACK1 plays an inhibitory role on Src and Fyn kinase's function [29,49,57,61,113,114]. Suppression of Src activity by RACK1 is central to the regulation of the growth of colon cells [114] and a new report suggests that by inhibiting Src activity, RACK1 stabilises E-cadherin and catenins at cell-cell contacts [115]. The release of RACK1 from Src is mediated by IGF-I [113], and the release of RACK1 from Fyn is mediated by activation of the cAMP/PKA pathway [56], resulting in the activation of both kinases (see also sections 5.3 and 6.2).

### RACK1 and transmembrane receptors

RACK1 interacts with cytoplasmic tails of several receptors including the Insulin-like Growth Factor Receptor I (IGF-IR), β-integrin receptor, the common beta-chain of the IL-5/IL-3/GM-CSF receptor [116], type I interferon receptor [117], inositol 1,4,5-trisphosphate receptors (IP3R) when at the cell membrane [118], the muscarinic M2 receptor [119], the adiponectin receptor [120] the androgen receptor [121], as well as several ion channels. As described below, the interaction of RACK1 with intracellular domains of transmembrane receptors results in the positive or negative regulation of receptor function.

### RACK1 and receptor tyrosine kinases

One of the best studied receptor tyrosine kinase (RTK)/RACK1 associations is the one between RACK1 and the IGF-IR [76,77]. RACK1 interacts with the IGF-IR in a wide variety of cells and the interaction site has been mapped to the C-terminus of the IGF-IR. RACK1 also interacts with the closely related Insulin receptor [77,122]. At the IGF-IR, RACK1 recruits a series of proteins including the adaptor proteins, Shc and IRS-1/2, the phosphatase, Shp2, and the transcription factor, STAT3 [77,113,122]. In doing so, RACK1 builds protein complexes to facilitate signaling downstream of the IGF-IR. RACK1 also binds the β subunits of integrin [123] and integrates and facilitates signaling between the IGF-IR and integrins during tumour cell migration [76,77,124]. This is mediated in part by the serine/threonine phosphatase PP2A as RACK1 binds PP2A and β1 integrin in a mutually exclusive manner [58,59]. In bridging the IGF-IR and β1 integrin into one complex, RACK1 facilitates the IGF-I and adhesion-mediated recruitment of other proteins to the complex to promote cell migration. Binding of RACK1 to integrins also promotes the engagement of survival signaling pathways [125], the MAPK pathway [126] and the recruitment of several PKC isoforms [127-129].

### RACK1 and ion channels

RACK1 is highly expressed in the CNS, mainly in neurons, during development and also throughout the adult brain, where it is localized to the neural cell bodies and dendrites [71,86]. In the CNS, RACK1 interacts with the cytoplasmic tail of ligand-gated and ion-gated channels and plays an important role in the regulation of the channels' functions. Specifically, RACK1 binds the cytoplasmic domain of the β1 and β3 subunits of the GABA_A _receptor (GABA_A_R) [96,130]. The interaction between RACK1 and the GABA_A_R plays an important role in facilitating the phosphorylation of the GABA_A_Rs by PKCβII, thereby modulating GABA_A_R function in response to the activation of muscarinic acetylcholine and the serotonin receptors [96,130,131].

RACK1 also plays an important role in regulating the function of the NMDAR. In the hippocampus and the dorsal striatum, RACK1 interacts with the cytoplasmic tail of the NR2B subunit of the NMDAR and simultaneously with the kinase, Fyn [49]. This simultaneous interaction allows Fyn to be localized in close proximity to its substrate, NR2B (Figure 5). However, as long as RACK1 is present in the complex, Fyn cannot phosphorylate the subunit [49]. Activation of the cAMP/PKA pathway leads to the dissociation of RACK1 from NR2B and Fyn [29,56]. Once RACK1 is removed from the NMDAR, Fyn is then free to phosphorylate NR2B, which in turn results in an increase in the activity of the channel [29,49,57] (Figure 5A). Interestingly, although the three proteins are expressed throughout the brain, the compartmentalization of Fyn to the NR2B subunit of the NMDAR by RACK1 is detected in some brain regions but not others. Specifically, the tri-molecular complex is detected in the hippocampus and dorsal striatum but is not found in the cerebral cortex or the ventral striatum [49,55-57] (Figure 5B, C). Furthermore, the compartmentalization of Fyn in close proximity to, or away from, the NMDAR bears functional consequences and provides the molecular mechanism underlying the difference in the sensitivity of the NMDAR to alcohol in these brain regions [55,57] (see also section 6.2).

Finally, RACK1 has been pulled out in a yeast 2-hybrid screen with the cytoplasmic tail of the large conductance Ca^2+ ^activated potassium (BK) channel [132]. RACK1 was shown to slow the activation of the channel [132], although the functional experiments were conducted in *Xenopus Oocytes *and must be confirmed in a more physiologically relevant system. Nevertheless, this study serves as an example of the ability of RACK1 to interact with ion-gated channels as well.

## RACK1 and Examples of Other Interacting Proteins

### RACK1 and the Androgen Receptor

RACK1 has been shown to interact with the androgen receptor (AR). In binding to the AR, RACK1 mediates translocation of the receptor into the nucleus [121], and also facilitates the cross-talk of the AR with other kinases [133]. Specifically, the recruitment of Src to AR-bound RACK1 facilitates the phosphorylation of the AR on tyrosine residues and regulates its transcriptional activity [133].

### RACK1 and 14-3-3

Very recently, using a proteomics strategy, we identified the scaffolding protein 14-3-3ζ as a binding partner of RACK1 (Neasta *et al*. 14-3-3/RACK1, in revision). We found that the interaction between the two scaffolding proteins is direct and does not require the prior phosphorylation of RACK1 on consensus phospho-14-3-3ζ binding sites. Using a RACK1-derived peptide array approach, we identified two putative binding sites for 14-3-3ζ located within the WD2-3 and WD4-5 propeller blade regions of RACK1. We found further evidence to suggest that 14-3-3ζ is necessary for the shuttling of RACK1 to the nucleus and to the subsequent activation of *BDNF *transcription in response to activation of the cAMP/PKA pathway (Neasta *et al*., 14-3-3/RACK1 in revision). These findings provide the first intriguing example that the interaction between two multifunctional scaffolding proteins, RACK1 and 14-3-3ζ, can provide a multiprotein signaling platform to tether signalosome.

### RACK1 and acetylcholinesterase

RACK1 interacts with the stress-induced splice variant of acetylcholinesterase (AChE-R) ([134]. The interaction between AChE-R and RACK1 leads to the recruitment of PKCβII in the CA1 and CA3 regions of the hippocampus, as well as the basolateral amygdala [134]. Furthermore, peripheral anionic site (PAS) inhibition of AChE in primary hippocampal neurons led to the redistribution of RACK1, decreased Fyn expression and NR2B insertion into the synaptic membrane, resulting in spontaneous excitatory post-synaptic currents (sEPSCs) [135].

## RACK1, Postranslational Modifications and Subcellular Compartmentalisation

### Post-translational modifications

In response to stress, RACK1 is sequestered into stress granules and inhibits apoptosis by suppressing stress-responsive MAPK pathways [136] Elegent studies carried out by Ohn *et al*, [137], have revealed that in response to arsenite-induced stress, RACK1 is reversibly modified by *O*-linked *N*-acetylglucosamine (O-GlcNAc) and moves from polysome-containing fractions to monosome-containing fractions of the cell. This study also indicates that when modified by O-GlcNAc, RACK1 moves to ribosomal subunit-containing fractions where it associates with the translational machinery and promotes stress granule assembly. Little is known about other post-translational modifications of RACK1 apart from phosphorylation which is emerging as an important factor that modulates the binding of proteins to RACK1. RACK1's sequence contains six tyrosine residues: Tyr-52 (located at the boundary of WD1 and WD2), Tyr-140 (WD 4), Tyr-194 (WD5), Tyr-228 and Tyr-246 (WD 6), and Tyr-302 (WD7). Phosphorylation of RACK1 on Tyr-52 by c-Abl mediates the interaction with FAK [124], which is required for cell adhesion and cell migration while phosphorylation/dephosphorylation of Tyr-302 in WD7 of RACK1 regulates the mutually exclusive association of RACK1 with PP2A and β1 Integrin [58,59]. Phosphorylation of RACK1 on Tyr-246 is required for the binding of Src kinase to RACK1 [138]. The interaction between RACK1 and Src is essential for the regulation of Src activity during the cell cycle [114,139]. RACK1 is also a substrate of Src. Specifically, Src phosphorylates RACK1 on Tyr-228 and strong evidence suggests that Src phosphorylates RACK1 on Tyr 246 also [48]. In addition, serine phosphorylation of RACK1 is also reported. Phosphorylation of RACK1 on Ser-146 promotes its dimerization which is required for the degradation of HIF-1α [140]. Taken together, these studies suggest that RACK1 is modified by signaling pathways which is important for protein binding and RACK1 location.

### Subcellular localization and translocation

RACK1 immunoreactivity can be detected in various cellular compartments including the cytosol, endoplasmic reticulum and the nucleus. In neurons, RACK1 is detected in cell bodies and dendrites but not in axons [71,86]. In addition, translocation of RACK1 from one subcellular location to another has been shown to mediate various cellular responses following a stimulus. For example, in response to PKC activation, RACK1 accompanies PKCβII to its site of action [60] (Figure 2), and as mentioned above, upon cellular stress induced by hypoxia or heat shock, RACK1 is sequestered into cytoplasmic stress granules preventing the activation of a stress-responsive MAPK pathway, which in turn leads to cell survival instead of cell apoptosis [136]. Moreover, the activation of the cAMP/PKA pathway in various types of cells, including neurons, induces RACK1 translocation to the nucleus [56,106,141,142] subsequently leading to the transcription of the brain-derived neurotrophic factor (*BDNF*) [56,57,106,143] and (Neasta *et al *14-3-3/RACK1 in Revision) (Figure 4). The RACK1 sequence does not contain known organelle localization motifs, or N-terminal lipid modification sites; nor does the sequence contain nuclear entry or nuclear export signals. In addition, there are no conclusive reports that the subcellular location of RACK1 is controlled by post-translational modification of RACK1 itself. Thus, the movement of RACK1 around the cell is most likely influenced by the particular cohort of proteins associated with RACK1 at any one time. An example, of such interacting protein is 14-3-3 which enables RACK1 to be transported to the nucleus in response to the activation of the cAMP/PKA pathway (Neasta et al 14-3-3/RACK1 in Revision).

### RACK1 in the nucleus

As detailed above, activation of the cAMP pathway in various types of cells, including neurons induces RACK1 translocation to the nucleus, subsequently leading to the transcription of several genes including *BDNF *[56,106,141,143] and (Neasta et al 14-3-3/RACK1 in Revision). In relation to *BDNF *transcription, using adenovirus-mediated siRNA delivery to knockdown the level of RACK1 in differentiated SHSY5Y cells and hippocampal neurons, we showed that activation of the cAMP pathway results in the association of RACK1 with the promoter IV region of the *BDNF *gene, leading to an increase in exon IV specific *BDNF *transcription [106]. RACK1 has been identified in several other complexes in the nucleus. It binds to the RQC domain and the acidic region of WRN and WRN may play an important role in retaining RACK1 in the nucleus [102]. Ki-1/57, a Ser/Thr protein kinase that interacts with chromo-helicase DNA-binding domain protein 3, forms a tight complex with RACK1 in the nucleus and treatment of cells with the PKC activator, PMA, mediates threonine phosphorylation of Ki-1/57 and disrupts the interaction with RACK1 [144]. Finally, RACK1 is also an essential component of systems required for regulating HIF-1α stability [54,145] and it does so by competing with HSP90 for binding to HIF-1α.

### RACK1 and ribosomes

An exciting development in RACK1 physiology is the realisation that RACK1 has a key role to play in the translation machinery [146]. The identification of RACK1 as a ribosome-associated protein was first made by mass spectroscopy [147], and in recent years RACK1 has become a well-established component of the ribosomal machinery through studies with ribosomes from yeast [83,84,89,148-151] and fungi [88], from unicellular algae [152] and plants [89,90,153-156] from protozoa [80] and mammals [85,86,137]. As discussed (Part 2), the RACK1 binding site, located on the 40S subunit of the eukaryotic ribosome, is now well defined by several structural studies.

Although ribosome-bound RACK1 might directly regulate translation *per se*, its function as a ribosomal protein is likely linked to its capacity to recruit a particular cohort of RACK1-associated proteins such as activated PKCβII. Indeed, PKC-mediated translational control has recently been shown to be dependent upon RACK1, and a RACK1-derived peptide that disrupts PKCβII binding to RACK1 or down-regulation of RACK1 with siRNA impairs PKC-stimulated translation [92]. The action of PKCβII in stimulating translation is thought to arise, at least in part, from its phosphorylation of eukaryotic initiation factor 6 (eIF6) bound to the 60S ribosomal subunit. Specifically, assembly of the ribosome by joining of 40S and 60S subunits is a rate-limiting step in translation, and PKCβII-mediated phosphorylation of a carboxy terminal serine (S235) in eIF6 triggers release of the initiation factor to facilitate this [91]. Identification of the ribosomal binding site for RACK1 has therefore suggested a model for ribosome assembly in which phosphorylation of eIF6 by PKC occurs on the 60S subunit as a result of an interaction with eIF6 and PKC bound to a nearby 40S subunit via RACK1 [84]. RACK1 is also known to bind eIF6 directly, although the loci on RACK1 for this interaction have not yet been defined and it is unclear whether phosphorylation of eIF6 by PKC involves simultaneous association of both enzyme and substrate initiation factor on the ribosome-bound RACK1.

In contrast to its role in promoting translation, other studies suggest that RACK1 may act at the ribosome to repress gene expression [148,157]. Specifically, very recently it has been shown that RACK1 interacts with components of the microRNA-induced gene silencing complex, contributing to recruitment of the complex to the site of translation and suggesting that RACK1 facilitates a post-initiation mode of miRNA-mediated gene repression [157]. RACK1 may also be involved in translation arrest, and its binding at the 40S subunit has been shown to be essential for nascent polypeptide-dependent translation arrest [158]. Finally, it is also feasible that the action of RACK1 at the ribosome may result in varying effects on gene expression by recruiting proteins that regulate translation of specific mRNAs in different ways. For example, Baum *et al*. suggested that recruitment of the mRNA-binding protein, Scp160p, to the yeast homolog (Asc1p) of RACK1 may influence the translation of specific mRNAs [159]. In addition to affecting the rate of translation of mRNAs, RACK1 is thought to play a role in influencing the location of translation, for example, in neurones [86].

## RACK1 and Physiological Functions

### RACK1 and development

In recent years, a critical role for RACK1 in development has been emerging. For example, in zebrafish, RACK1 affects membrane localization of van gogh-like 2 (Vangl2), and the Vangl2-interacting region of RACK1 has been shown to exert a dominant-negative effect on Vangl2 localization and gastrulation [160]. The interaction between RACK1 and tyrosine-protein kinase-like 7 (PTK7) has also been shown to be a requirement for neural tube closure in *Xenopus *[161]. In *Arabidopsis*, where three RACK1 homologs are present, the RACK1 gene products are essential regulators of plant development [162,163]. RACK1 homologs in *Drosophilia *[164], *Aspergillus nidulans *[165], *Schizosaccharomyces pombe *[166] and *Trypanosoma brucei *[167] have similarly been shown to be central to various stages of the developmental process.

### RACK1 and circadian rhythm

Recent elegant studies by Robles *et al*. [103] have identified RACK1 as an integral component of the mammalian circadian clock. Their findings suggest that RACK1 and PKCα regulate CLOCK-BMAL1 transcriptional activity [103]. Their findings also indicate that the mechanism of action of RACK1 and PKCα are intrinsically linked and that the function of one is dependent on the other. Although it is well established that RACK1 regulates PKC location [60,92,134,142], this exciting development in RACK1 biology reveals a novel mechanism of action.

### RACK1 and cell migration

The scaffolding of signaling proteins by RACK1 at receptors is particularly important in dynamic processes such as cell migration, cell adhesion and cell spreading [76,77,113,124]. All of these processes require the highly regulated converging of transient signaling between receptors. For example, RACK1 was first found to be a mediator of cell spreading by establishing contact with the extracellular matrix and growth factor receptors at adhesion sites [76].

**Figure 10:**
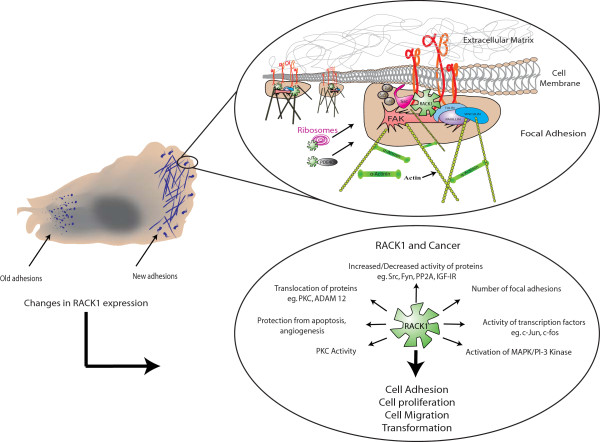
**RACK1 is an important regulator of Focal Adhesion assembly**. When a cell is in contact with the Extracellular Matrix, integrins are clustered on the cell surface. This clustering of integrins leads to the recruitment of FAK and to the establishment of focal adhesions. At focal adhesions, RACK1 recruits key structural proteins, kinases and phosphatases to help nurture the developing focal adhesion. It is likely that at focal adhesions RACK1 brings in phosphodiesterases and ribosomes which facilitate local signaling and translation. A change in RACK1 expression (both up and down) has consequences for the activity and stability of RACK1-associated proteins and is believed to be a contributing factor in the development and progression of cancer.

The role of RACK1 as a scaffolding protein is clearly evident during cell migration. Cell migration is a fundamental process required for embryonic development, wound healing and immune responses, and the components of cell migration are functionally conserved in evolution. The failure of cells to migrate, or indeed their migration to the wrong location, gives rise to serious life threatening consequences such as defective brain development, a malfunctioning immune system and leads to the materialization of cancer. Understanding cell migration presents a considerable challenge because it results from the coordinated activity of several individual intra- and extracellular component processes. These processes include several signaling pathways, and require significant and well-orchestrated cross-talk between cell surface receptors and elements of the cell cytoskeleton. RACK1 is essential for cell migration, and the protein binds to many components of the cell migration machinery including kinases, phosphatases and the cytoplasmic domains of cell surface receptors [46,47]. RACK1 is located in areas of cell protrusion that are rich in paxillin [168,169] and can increase the phosphorylation of FAK [169]. The interaction of RACK1 with β1 and β2 integrins and Src regulates cell adhesion and cell spreading [123,168]. RACK1 has been reported to bind to components of the cell cytoskeleton [128,170]. The identification of proteins such as these as RACK1 partners has led to the establishment of RACK1 as a key regulator of Focal Adhesion assembly (Figure 10). Focal adhesions are large dynamic macromolecular assemblies with both mechanical components and cell signaling components [171-175]. Specifically, focal adhesions are the mechanical link between the cell and the extra cellular matrix (ECM), that are formed after integrins are clustered on the cell surface. Integrin clustering is sufficient to promote the phosphorylation of FAK on Tyr-397. This in turn generates a binding site for the Src homology 2 (SH2) domain of Src Family protein tyrosine kinases (Src-family PTKs). The recruitment of Src-family PTKs to FAK is dependent on the initial autophosphorylation of Tyr-397 and results in the phosphorylation of FAK at secondary sites to ensure full activation of FAK. This correlates with an increase in kinase activity and the ability of FAK to cooperate with multiple signaling pathways through its direct interaction with other non-receptor tyrosine kinases, cell surface receptors, cytoskeletal proteins and other adaptor proteins. FAK is widely regarded as being the convergence point of growth factor and integrin signaling [176,177], yet how FAK phosphorylation is regulated within the growth factor and integrin network is not fully understood. Nevertheless, the phosphorylation status of FAK clearly regulates cell adhesion and cell migration in response to growth factors, integrin stimulation and components of the extracellular matrix. RACK1 is essential for FAK's function as it scaffolds the kinase for activation by both IGF-I and adhesion signals [124] and the binding locus for FAK on RACK1 has been mapped to Tyr-52 in WD2 of RACK1 [124] and the interaction site of RACK1 on FAK was subsequently identified [112]. Suppression of RACK1 expression disrupts FAK activity, cell adhesion and cell spreading [113,124,169], two essential steps in the cell migration process, by the interaction of RACK1 with FAK and by the ability of RACK1 to regulate FAK activity. In addition, recent studies suggest that attenuation of RACK1 expression suppresses VEGF-mediated cell migration by disrupting the path between VEGF, the PI3K pathway and Rac1 [178]. RACK1 (and RACK1A) is known to interact with several components of the Rac1 complex that are involved in cell migration [179,180]. Cell migration is dependent on the response to chemoattractant gradients, and in Jurkat cells RACK1 interacts with Gβγ at the leading edge of cells [181]. In this system, RACK1 inhibits cell migration and overexpression of full length RACK1 or truncation mutations that retain the Gβγ binding, actually inhibit cell migration [181]. There are other reports suggesting that RACK1 inhibits cell migration [128] but under most circumstances, RACK1 facilitates cell migration. A large part of what RACK1 does in cell migration, at least in adherent cells, is mediated by its ability to bind to a series of kinases and phosphatases that affect the phosphorylation and subsequent activity of other RACK1 binding partners or proteins in complexes where RACK1 is found. RACK1 interacts with several PKC isoforms during cell migration including PKCβ [170] and PKCε [127]. Activation of the PKC signaling pathway also enhances the interaction between RACK1 and Src [138]. Src tyrosine kinase phosphorylates RACK1 and phosphorylation of RACK1 is required for Src binding to RACK1 via its SH2 domain [48]. Src is a well-known regulator of cell adhesion, cell spreading and cell migration [61,62,114,168]. Src activity is inhibited by the binding of RACK1 but RACK1 can transport Src to specific cellular compartments where Src can function. In fibroblasts and epithelial cells, the interaction between RACK1 and Src is disrupted by IGF-I stimulation of the IGF-IR signaling pathway [113]. This results in the release of now active Src at a specific location and is an example of how RACK1 can regulate the local activation of proteins. The regulation of Src activity by RACK1 is also required for modulating paxillin during cell migration [182], for regulating Sam68 and p190RhoGAP signaling [183], to control cell protrusion during cell migration [168] and for regulating cell cycle checkpoints [62,114,139]. The dynamic interaction between RACK1 and Src is also required for regulation of the phosphorylation and function of the Androgen receptor [133]. There are now data suggesting that the interaction between RACK1 and Src goes beyond regulating cell adhesion and cell migration and Mamidipudi *et al*, have shown that the interaction has a pro-apoptotic function [184]. The interaction of RACK1 with phosphatases during cell adhesion is equally important and mounting evidence suggests that RACK1 receives signals to recruit phosphatases that dismantle specific signaling pathways and cytoplasmic support systems such as focal adhesions [58,124,185]. In IGF-IR signaling, RACK1 promotes IGF-I-mediated cell migration by regulating the formation of a RACK1-IGF-IR and β1 integrin complexes, which is mediated by the mutually exclusive association of RACK1 with PP2A and β1 integrin [58,59]. In pancreatic cells, RACK1 and PP2A form a complex with IRE1α in a signaling pathway that regulates insulin production [186]. In addition, RACK1 and phosphatases are involved in cell migration in other systems. Fo example, in the retina, retinal ganglion cell axons are guided by signaling pathways that are regulated by an interaction between RACK1 and PTP μ [185,187-189].

## RACK1 in Disease

Since its discovery, RACK1 has been associated with various aspects of diseases. Alterations in RACK1 homeostasis is associated with brain developmental disorders [161], heart failure [99], pulmonary arterial hypertension [190], renal failure [191], muscle atrophy [192] and dysfunctional sperm development [193], as well as cancer and addiction (detailed in sections 6.1 and 6.2). In addition, altered RACK1 expression has negative consequences for the properties and activity of RACK1 binding partners. The association of RACK1, PKCβII and a variant of the acetylcholinesterase AChE has been implicated in stress-induced behaviors [134] (see also section 6.2). Increased RACK1 expression has also been observed in the frontal cortex of patients with bipolar disorder [194], while decreased RACK1 expression is reported in the cortex of Down syndrome patients [74]. In addition, RACK1 levels are significantly reduced in aged rats [195], and decreased RACK1 expression is reported in post-mortem brains of Alzheimer's disease (AD) patients [72,73]. However these changes were not found in another study, which compared the levels of RACK1 in AD patients and age-matched controls [196]. Nevertheless, the pathophysiology of AD patients also highlights defective PKC activity and localisation which correlates with decreased RACK1 expression [197]. PKC and muscarinic receptor signaling are intrinsically linked, and amyloid-β impairs the regulation of PKC and GABAergic signaling. Overexpression of RACK1 has been shown to restore the regulation of these processes [198].

Several reports suggest a role for RACK1 in the immune system. Specifically, studies carried out in T Jurkat cells indicate that RACK1 regulates directional cell migration [181] while other reports suggest that RACK1 regulates T cell apoptosis [199]. In Rice, RACK1A functions in innate immunity by interacting with the GTP form of Rac1 [179] and having a central role in the production of reactive oxygen species in response to infection. Because of its role in several signaling pathways, it is not surprising, therefore, that RACK1 is hijacked by viruses to facilitate viral replication [200-202].

### RACK1 and cancer

Many reports indicate that RACK1 plays an important role in cancer progression and that its expression is altered during angiogenesis and in many human carcinomas. Specifically, RACK1 expression is being assessed as a prognostic indicator in breast cancer [203,204], where increased RACK1 expression is strongly related to advanced clinical stage [205]. In pulmonary adenocarcinomas, increased RACK1 expression is associated with pathological stage and tumor size and is also a potential diagnostic marker [206]. In contrast, some reports suggest that RACK1 expression is reduced in breast cancer [207]. In addition, RACK1 may protect cells from apoptosis in breast cancer [204,208]. RACK1 is upregulated in hepatocellular carcinoma where it increases the translocation of A Disintegrin And Metalloprotease 12 (ADAM12) to the plasma membrane in response to PKC activation [209] and it is upregulated in metastatic melanoma, where it amplifies PKCα and PKCβ activity [210]. In melanoma, constitutive activation of the MEK-ERK signaling pathway leads to elevated c-Jun activity and increased the transcription of RACK1 [211]. RACK1 is also implicated as a key player in ovarian cancer [212], prostate cancer [188] and in cancers caused by pathogens such as human papillomavirus 16 (HPV 16) [213] and *Helicobacter pylori *[214].

There are several mechanisms by which RACK1 contributes to the progression of cancer. Firstly, RACK1 is upregulated during angiogenesis [215] where it promotes activation of the PI3K/Akt/Rac1 pathway [178] and regulates HIF-1 transcriptional activity [216,217]. RACK1 also plays an important role in cell proliferation, cell adhesion and cell migration, which are all essential components of transformation as described in section 5.3. The interaction between RACK1 and FAK is constitutive and FAK phosphorylation on the RACK1 scaffold is mediated by the IGF-IR [124]. The interaction between RACK1 and FAK is found at nascent adhesions and is required at the leading edge of polarized cancer cells to sense direction. RACK1 also brings FAK and PDE4D5 together which strongly suggests that adhesion signaling and cAMP signaling are linked in cancer [111,112]. At adhesion sites, RACK1 recruits paxillin [168,169], integrins and Src to regulate cell adhesion and cell spreading [113,123,124,168], β-tubulin [218] and other components of the cell cytoskeleton [170]. Thus, in cancer, RACK1 becomes a focal point for transformation signaling (Figure 10). RACK1 recruits ribosomes and PKC (discussed above) and scaffolds central components of the MAPK pathway [126] and the PI3K pathway [76,77,113]. As well as this, RACK1 scaffolds a host of other different kinases and phosphatases, and the activity of several of these proteins is altered in cancer. All of these proteins, signaling pathways and protein complexes require tight regulation. Subtle changes in the protein expression (both up and down) of a fundamental protein such as RACK1 can be expected to have dramatic consequences on the regulation of these key pathways and therefore on the development and progression of neoplastic disease.

### RACK1 and addiction

Addiction is a costly, devastating psychiatric disorder. The disease manifests itself in the intake of the drug regardless of the negative consequences, and is associated with tolerance, dependence, withdrawal, relapse and craving [219]. Altered levels and compartmentalization of RACK1 was reported by us and others to be linked to exposure to drugs of abuse. Specifically, changes in RACK1 levels have also been described in response to the treatment of rodents acutely and chronically with morphine. Escriba *et al*. found that acute treatment of rats with morphine results in increased RACK1 levels in the frontal cortex, whereas chronic treatment with morphine led to a decrease in the level of RACK1 in this brain region [220]. The authors also report that changes in RACK1 levels paralleled with changes in the levels of PKCα and PKCβ [220]. In contrast, Wan *et al*. found that chronic treatment of mice with morphine led to an increase in the level of RACK1 in this brain region [221]. Finally, Liu *et al*. reported that alteration of RACK1 levels result in changes in the rewarding properties of morphine [222,223]. In summary, although changes in RACK1 expression are linked to morphine administration, further studies are needed before any conclusions can be drawn.

In addition, over the past several years, we have demonstrated that one of the molecular mechanisms by which alcohol can simultaneously alter several signaling pathways is by altering the cellular compartmentalization of RACK1. Furthermore, we obtained data to suggest that the functional outcomes of the altered localization of RACK1 in the presence of alcohol are diverse, as RACK1 contributes both to mechanisms that underlie but also those that prevent the escalation of behaviors that underlie alcohol addiction. The interest in the potential role of RACK1 in alcohol addiction was initiated with the observation that exposure of several cell lines as well as primary neurons to alcohol results in a dose- and time-dependent translocation of RACK1 to the nucleus [141-143,224]. The nuclear translocation of RACK1 in response to alcohol was shown to be dependent on the activation of the cAMP/PKA pathway [141]. Interestingly, prolonged incubation of cells with alcohol (12-48 hrs) results in the gradual distribution of RACK1 out of the nucleus, which was resistant to an acute challenge of alcohol [143,224]. These results suggest a cellular alcohol tolerance leads to altered compartmentalization of RACK1. Furthermore, as a result of the nuclear localization of RACK1 in response to acute alcohol exposure, the translocation of active PKCβII is inhibited [142], and the transcription of several genes are induced [141-143,224].

Of great interest is the observation that the expression of the growth factor BDNF is increased in response to alcohol-mediated RACK1 nuclear localization [143]. Importantly, molecular and behavioral studies suggest that RACK1 and BDNF are part of an endogenous homeostatic pathway that delays or prevents the development of alcohol addiction. Specifically, acute increase of RACK1 protein levels in hippocampal and striatal neurons using the Tat protein transduction approach (Tat-RACK1), led to increases in the levels of BDNF and a similar increase in BDNF expression was observed after systemic injection of the Tat-fusion protein in mice [143]. The systemic administration of mice with Tat-RACK1 resulted in a robust decrease in alcohol but not water consumption in mice in a BDNF-dependent manner [143,225]. Specifically, the activity of Tat-RACK1 on alcohol consumption was reduced in BDNF heterozygote mice as compared to wild-type mice, and, systemic administration of a BDNF receptor TrkB inhibitor increased alcohol consumption in wild-type controls, but not in BDNF heterozygote mice [143,225]. We also provide data that the inhibitory actions of RACK1 on alcohol intake are localized to the dorsal striatum as infusion of Tat-RACK1 directly into the dorsal striatum of rats reduced rat operant self-administration of alcohol [225]. Finally, we obtained data to suggest that the consequence of RACK1 mediated increase in BDNF expression in response to alcohol is the increase in the expression of the dopamine D3 receptor and Dynorphin which in turn mediate the reduction in the sensitivity of rodents to alcohol [225,226]. Together, these results suggest that RACK1 inhibits alcohol consumption by increasing the expression and secretion of BDNF, and that secreted BDNF counteracts the reinforcing or rewarding effects of alcohol. Thus, the RACK1 and BDNF pathway contributes to cellular and molecular processes that influence the motivation to seek alcohol.

Interestingly, the altered compartmentalization of RACK1 in response to alcohol exposure in specific brain regions also contributes to neuroadaptations that underlie the development of alcohol addiction. As described in section 1.2, in the hippocampus and the dorsal striatum but not in the cerebral cortex or ventral striatum, RACK1 localizes Fyn in close proximity to the NR2B subunit of the NMDAR, but inhibits the ability of Fyn to phosphorylate the channel until the appropriate signal occurs [29,49,55,57,141] (Figure 5A). This specific compartmentalization of Fyn to the NMDAR complex via RACK1 has broad implications for the actions of alcohol on the channel. In the hippocampus, alcohol treatment results in the release of RACK1 from the NMDAR complex in a mechanism that depends on the activation of the cAMP/PKA signaling cascade [57]. The dissociation of the tri-molecular complex in response to alcohol enables Fyn to phosphorylate NR2B [57]. These molecular changes in the hippocampus lead to a reduction in the inhibitory actions of alcohol on channel activity, in rebound potentiation of the activity of the channel when alcohol is washed out, and to the development of acute desensitization to the inhibitory actions of alcohol on the activity of the channel [57]. In the dorsal striatum, RACK1 dissociation from Fyn and NR2B and the phosphorylation of the subunit by Fyn leads to long-lasting long-term facilitation of the activity of the channel [55]. Importantly, this biochemical neuroadaptation in the dorsal striatum is part of a mechanism that underlies excessive alcohol consumption and relapse [55,227].

In summary, the altered compartmentalization of RACK1 in the presence of alcohol in specific brain regions results in diverse physiological consequences that both contribute to, and prevent, the development of behaviors associated with alcohol addiction.

## Summary and Future Perspectives

Since its identification as an anchoring protein for active βIIPKC [42], the number of binding partners and functions for RACK1 have increased exponentially. It is intriguing that a single protein can be involved in such a large number of diverse biological functions. One possibility is that the different intracellular compartmentalization of RACK1 in different cell types and regions allows the scaffolding protein to interact with multiple proteins and to regulate various functions in a cell specific manner (see Figure 10 as an example). Another possibility to explain the multiple functions of RACK1 is that although the protein itself is ubiquitously expressed, its binding partners are not. For example, RACK1 is ubiquitously expressed in the adult mouse brain, however, a much more restricted expression pattern is observed for PKCβII [71,228]. Interestingly, as described above, RACK1 can be associated with different binding partners in different brain regions. Specifically, although similar type of neurons are found in the hippocampus and cortex as well as in the ventral and dorsal striatum, RACK1 is associated the NR2B subunit of the NMDAR in the hippocampus and the dorsal striatum, [49,55-57]but not cerebral cortex but not in the ventral striatum [55,57]. These findings suggest that RACK1 scaffolds different proteins in these brain regions. The reasons for cell type or brain region specificity of RACK1 association with its binding partner is unclear and therefore it would be of great interest to explore this further.

Furthermore, we now know that proteins, such as PP2A and β1 integrin, compete for binding to RACK1 [58,59], and that phosphorylation of RACK1 on tyrosine and serine residues is critical for the way in which RACK1 functions during cell adhesion and cell migration [61,114,124]. It is thus essential that we gain a better understanding of what determines the hierarchy of protein binding to RACK1 and what signals orchestrate complex formation on RACK1. For example, growth factor and adhesion signaling converge on RACK1 [76,113,122,124], but it of interest to now focus on how other external environmental cues dictate the binding profile and cellular distribution of RACK1 and how RACK1 integrates these signals with such a high degree of specificity and regulation.

Finally, the unique structure of RACK1 and the expansion of knowledge of the protein-protein interactions mediating the binding of RACK1 to partners provide us with a great opportunity to develop new therapeutics by the design and development of peptides, and in the future possibly small organic compounds, which will interfere with specific binding of proteins to RACK1. This will permit subtle manipulation of the activity of specific enzymes, receptors and signaling pathways.

## Competing interests

The authors declare that they have no competing interests.

## Authors' contributions

DA, DR and PK contributed equally to the writing of the manuscript. All authors read and approved the final version of the manuscript.

## Endnotes

### Footnote 1

^1^A convenient graphical representation of consensus residues at particular sites within WD-repeats has been given by Xu and Min [6]. Significant diversity is seen across repeats in WD40 proteins and we refer to 'typical' repeats as ones that preserve the core interactions summarised in Figure 7.
